# The proportion, species distribution and dynamic trends of bloodstream infection cases in a tertiary hospital in China, 2010–2019

**DOI:** 10.1007/s15010-021-01649-y

**Published:** 2021-06-28

**Authors:** Jiewei Cui, Meng Li, Jiemin Cui, Juan Wang, Xiaofei Qiang, Zhixin Liang

**Affiliations:** 1 Department of Pulmonary and Critical Care Medicine, Chinese PLA General Hospital, Fuxing Road No. 28, Beijing, 100853 China; 2 Department of Hematology, The First Medical Centre of Chinese PLA General Hospital, Fuxing Road No. 28, Beijing, 100853 China; 3 Department of Radiology, Shenxian People’s Hospital, Xingfu Road No.303, Liaocheng, 252400 China; 4 Medical Team of Helicopter Unit, The Second CAPF General Mobile Force, Deyang, 618100 China

**Keywords:** Bloodstream infection, Proportion, Species distribution, Time trend, Cochran–Armitage trend test

## Abstract

**Background:**

Recent epidemiological studies on bloodstream infection (BSI) that include the proportion, species distribution and dynamic changes are scarce in China. This study was performed to understand these epidemiological data of BSI over the past 10 years in China.

**Methods:**

Using a prospective nosocomial infection surveillance system, this study was retrospectively performed in one of the largest hospitals in China. The time trend was tested using the Cochran–Armitage trend test in R Programming Language.

**Results:**

From 2010 to 2019, there were totally 9381 episodes of BSI cases out of 1,437,927 adult-hospitalized patients in the hospital, the total proportion of BSI cases was 6.50‰ (6.50 episodes per 1000 adult-hospitalized patients) and the proportion had significantly decreased (8.24–6.07‰, time trend *P* < 0.001). Among the 9381 episodes of BSI, 93.1% were bacteremia and others were fungemia (6.9%). As the most common species, the composition ratios of coagulase-negative *staphylococcus* (25.6–32.5%), *Escherichia coli* (9.8–13.6%) and *Klebsiella pneumoniae* (5.3–10.4%) had been dynamically increased (all time trends *P* < 0.05) and the proportion of *Pseudomonas aeruginosa* had decreased (4.0–2.4%, time trend *P* = 0.032). However, *Staphylococcus aureus* (3.3–3.1%) and *Acinetobacter baumannii* (4.4–4.2%) had not changed significantly (*P* > 0.05). These common species were consistent with China Antimicrobial Surveillance Network reported in 2018 (2018 CHINET report), but their composition ratios were different. In addition, among bacteremia, the proportion of multidrug-resistant bacteria gradually increased from 52.9 to 68.4% (time trend *P* < 0.001).

**Conclusion:**

The proportion and species distribution of BSI were dynamically changing along certain trends. These trends deserved more attention from clinicians and researchers.

**Supplementary Information:**

The online version contains supplementary material available at 10.1007/s15010-021-01649-y.

## Introduction

Bloodstream infection (BSI) refers to various pathogenic microorganisms that have invaded the blood, primarily bacteria and fungi [1]. With the increasing number of invasive procedures and the unreasonable use of broad-spectrum antibacterial drugs and corticosteroids, the incidence and mortality of BSI increase annually, as has been reported in many studies [2, 3]. For example, the proportion of BSI cases increased by 64% from 945 to 1,546 per 100,000 hospitalized patients from 2000 to 2013 in a Swedish county, and the 30-day mortality of BSI was up to 12.8% [4].

The administration of early empirical antimicrobial therapy based on the epidemiology of the species distribution and its antimicrobial susceptibility is crucial. Previous studies revealed that Gram-positive bacteria were more prevalent than Gram-negative bacteria in BSI among adult and pediatric hematology and cancer patients in the United States and most European countries, while the Gram-negative bacilli accounted for the majority of BSI (54%) in Colombian hospitals [5, 6]. However, the clinical microbiological data of BSI have constantly changed in recent years [6, 7]. For example, the proportion of Gram-negative bacteria increased from 44 to 53% over 9 years and that of Gram-positive bacteria decreased from 49 to 45% in an Australian tertiary hospital [8].

There were many studies on BSI in China, but most of these studies were separate cases studies involving the clinical characteristics, the risk factors of these BSI cases and (or) the species distributions and drug resistance of BSI. For example, Liu J et al. found that the 30-day mortality of carbapenem-resistant *Klebsiella pneumoniae* bacteremia was 24.7% and that the only risk factor was carbapenem exposure within 30 days before the onset of bacteremia in 89 oncohematological patients (HR 25.122) [9]. Bai Y et al. collected 174 patients with BSI after interventional therapy from 2013 to 2018 and found that Gram-positive bacteria accounted for 56.05% of BSI in these patients and that coagulase-negative Staphylococcus was the main infectious bacteria [10]. They also found that days of prophylactic antibiotic use (OR 1.586, *P* < 0.05) and replacement of antibiotics (OR 13.349,* P* < 0.05) were the main risk factors associated with the development of BSI using multivariate analysis.

Nonetheless, due to the complexity and huge financial expense of BSI surveillance, recent epidemiological studies on BSI in China that include the proportion, species distribution and dynamic changes are scarce. To understand the changing trends in the epidemiology of BSI, the present study retrospectively analyzed the proportion, species distribution and drug resistance of BSI and their dynamic changes over 10 years in one of the largest tertiary hospitals in China, Chinese People's Liberation Army General Hospital (CPLAGH). This hospital is one of the largest general hospitals and integrates medical treatment, teaching and scientific research. CPLAGH primarily provides medical services for all military and nonmilitary patients in northern China, and there were more than 140,000 hospitalized patients per year. Therefore, to a certain extent, the dynamic trends of the microbiology and proportion of BSI in this hospital might basically reflect the epidemiology of BSI in tertiary hospitals in northern China.

## Methods

### Hospital setting

This retrospective study was performed in the Chinese People’s Liberation Army General Hospital (CPLAGH). All data were collected by reviewing the prospective real-time nosocomial infection surveillance system (RT-NISS) medical electronic database in the hospital. The system is capable of daily, automatic, and prospective screening of all suspicious infections of hospitalized patients, such as BSI, respiratory tract infection, urinary tract infection, surgical site infection, gastrointestinal tract infection, skin and soft tissue infection and other infections. Simultaneously, the system also can eliminate contamination and colonization infections. A previous study showed that the sensitivity and specificity of the RT-NISS system were 98.8% and 93.0%, respectively, for nosocomial infections [11].

Blood was cultured with the BacT/ALERT® 3D™ system (Becton–Dickinson, Sparks, MD, USA) in the microbiology laboratory. Species were identified and then in vitro antibiotic susceptibility was determined with Vitek II (bioMérieux, France).

### Case definition

#### Positive-blood culture and the definition of BSI

Among the hospitalized patients in our hospital, blood cultures are received based on clinical diagnosis and treatment. Only patients who are suspected of BSI by clinicians will receive blood cultures. For example, patients will receive blood culture only when they have symptoms and signs related to BSI such as fever, chills, and unexplained hypotension. Patients who are not suspected of BSI do not need to receive blood cultures. All suspicious BSI were screened and under surveillance by the RT-NISS system. The following screening strategies for BSI were used. ① At least one positive blood culture: each set of blood cultures in our hospital included an aerobic blood culture bottle and an anaerobic blood culture bottle. A positive blood culture was defined as at least one isolation of microorganisms for a set of blood cultures, including bacterial, fungal and other rare pathogens. ② At least one BSI-related clinical symptom, such as chills, fever (> 37.3 ℃), and hypotension (systolic pressure < 100 mmHg). ③ Elevated levels of at least one BSI-related molecular marker, such as procalcitonin (PCT), C-reactive protein (CRP), and white blood cell count (WBC).

All hospitalized patients with positive blood cultures were considered as being suspicious for BSI, but only patients who had at least one BSI-related clinical symptom and (or) elevated levels of at least one BSI-related molecular marker were diagnosed with BSI by the RT-NISS system. Otherwise, the positive blood culture was considered a contaminant or colonization.

#### Inclusion and exclusion of BSI

The RT-NISS system identified repeated and identical BSI in the same patient. If the patient who was diagnosed with BSI had two or more positive blood cultures caused by an identical specie within 14 consecutive days, only the first blood culture was used to diagnose an episode of BSI, and the other cultures were excluded. However, if the identical BSI recurred after 14 days, the recurrent BSI was considered a new episode of BSI by the RT-NISS system. In addition, if the patient had one or more positive blood cultures caused by two or more different species and each of the species in the blood culture could be diagnosed as BSI, then each species that caused the BSI was considered as a separate episode of BSI. A BSI caused by coagulase-negative staphylococci (CoNS) or other potential skin contaminants was considered to be significant when the same species grew in at least two blood cultures within a 48-h period.

#### Community-acquired BSI and hospital-acquired BSI

BSI was divided into community-acquired BSI (CA-BSI) and hospital-acquired BSI (HA-BSI) based on the onset date and the date when the positive blood culture was drawn. The BSI was defined as a CA-BSI when its onset was within 48 h after admission, and HA-BSI was defined when BSI onset occurred longer than 48 h after admission.

### Study variables

From January 1, 2010, to December 31, 2019, the total number of adult-hospitalized patients diagnosed with BSI each year and the total number of adult-hospitalized patients were collected for each year. The proportion in each year and the dynamic trend were calculated.

The following microbiological data were collected: the species of the microorganism isolated in the blood culture; the gram stain type of the species that led to bacteremia (defined as Gram-positive cocci, Gram-negative bacilli and others); the species that led to fungemia (defined as Candida or non-Candida); the antibiotic resistance of bacteria (defined as multidrug resistance or sensitive according to the Clinical and Laboratory Standards Institute (CLSI) standards); and the corresponding numbers. The composition ratios were separately calculated annually; then, their dynamic time trend could be evaluated from 2010 to 2019. Epidemiologically significant antimicrobial categories were constructed for each bacterium. Lists of antimicrobial categories proposed for antimicrobial susceptibility testing were created using documents and breakpoints from the annually updated Clinical Laboratory Standards Institute (CLSI), such as CLSI 2010 edition (M100-S20) [12] and CLSI 2017 edition (M100-S27) [13]. MDR was defined as acquired non-susceptibility to at least one agent in three or more antimicrobial categories (such as aminoglycosides, erythromycins, penicillins, cephalosporins, β-lactams, carbapenem, tetracyclines, glycopeptides, linezolid, and sulfa antibiotics), but not three agents of the same antimicrobial category [14]. Penicillins, cephalosporins and carbapenems were considered as 3 ‘classes’ of different antibiotics.

The dynamic time trends of the proportion and species distribution in these 10 years were the focus of the present study. To reduce the coefficient of variation of all the variables in one year, the average value of each variable over two consecutive years was used to assess dynamic changes. Therefore, the 10-year data were divided into five groups: 2010–2011, 2012–2013, 2014–2015, 2016–2017 and 2018–2019.

### Statistical analysis

The statistical analysis was performed using SPSS version 22.0 software (SPSS, Inc., Chicago, IL, USA) or the R Programming Language (version 3.5.3). The number of adult-hospitalized patients and the episodes of BSI were described as N, and the proportions of BSI among all adult-hospitalized patients were described as N ‰ (N episodes per 1000 adult-hospitalized patients). The number of species and the composition ratios were described as *N* and a percentage (%), respectively. The dynamic time trends of the proportion of BSI and the composition ratios of the various species were statistically analyzed using the Cochran–Armitage trend test in R Programming Language (‘CATT’ package), and the 'CATT' package was exhibited as “Supplemental Material.R”. Although the Cochran–Armitage trend test was used to statistically analyze these time dynamic trends, the Mantel–Haenszel Chi-square test by the SPSS software is approximately equivalent to the Mantel–Haenszel Chi-square test and could be also used in this research. In addition, the Pearson correlation coefficient (*R*) were used to exhibit the directions of these trends (increase or decrease). All results with a 2-tailed *p* value < 0.05 were significant. *R* > 0 represented that the dynamic time trend increased, and *R* < 0 represented that the dynamic time trend decreased.

## Results

### The proportion of BSI and dynamic time trend

**Fig. 1:**
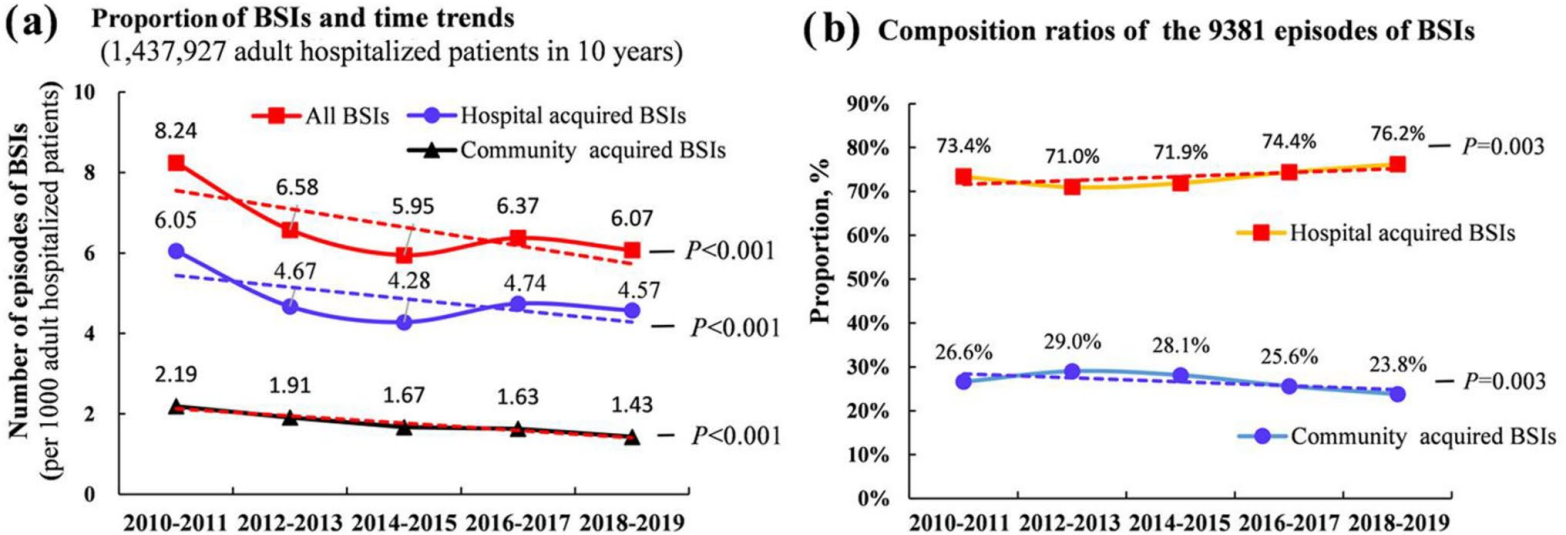
The proportion and the composition ratios of the 9381 episodes of bloodstream infection (BSI) and their dynamic time trends from 2010 to 2019. The dashed straight lines represented the time trends and were used to visually show the direction of the dynamic trends (increase or decrease). *P* < 0.05 represented the direction of the dynamic trends were significant. The results showed that the proportion of BSI among the adult-hospitalized patients had significantly decreased from 2010 to 2019. However, among the 9381 episodes of BSI, the composition ratio of the hospital-acquired BSI (HA-BSI) had increased significantly

From January 1, 2010, to December 31, 2019, there were 1,437,927 adult-hospitalized patients in the hospital and 9381 episodes of BSI. The total proportion of BSI among the adult-hospitalized patients over these 10 years was 6.50‰ (6.50 episodes per 1000 adult-hospitalized patients). As shown in Table 1 and Fig. 1a, although the number of adult-hospitalized patients and the number of BSI over the two consecutive years increased from 2010 to 2019, the time trend of the average proportions of BSI decreased gradually from 8.24 to 6.07‰. The Cochran–Armitage trend test was statistically significant (*P* < 0.001). Both the proportions of community-acquired BSI (CA-BSI) and hospital-acquired BSI (HA-BSI) among all adult-hospitalized patients in this hospital showed similar trends. The CA-BSI decreased from 2.10 to 1.43‰, and the HA-BSI decreased from 6.05 to 4.57‰. However, as showed in Fig. 1b, among the 9381 episodes of BSI, the composition ratio of the HA-BSI increased significantly from 73.4% (1279/1743) to 76.2% (1664/2184). In contrast, the composition ratio of the CA-BSI decreased significantly from 26.6% (464/1743) to 23.8% (520/2184). These time trends were statistically significantly (time trend *P* = 0.018).

**Table 1:** The proportion and the dynamic trend of BSI in adult-hospitalized patients

	Total	2010–2011	2012–2013	2014–2015	2016–2017	2018–2019	*Z* value	*P* value	*R*
Number of hospitalized patients	1,437,927	211,564	257,621	287,033	322,162	359,547			
Number of episodes of BSI	9381	1743	1694	1707	2053	2184			
Proportion of BSI (‰)	6.52‰	8.24‰	6.58‰	5.95‰	6.37‰	6.07‰	−6.039	< 0.001*	−0.007
Hospital-acquired BSI	6901 (4.80‰)	1279 (6.05‰)	1202 (4.67‰)	1228 (4.28‰)	1528 (4.74‰)	1664 (4.63‰)	−5.253	< 0.001*	−0.005
Community-acquired BSI	2480 (1.72‰)	464 (2.19‰)	492 (1.91‰)	479 (1.67‰)	525 (1.63‰)	520 (1.44‰)	−4.195	< 0.001*	−0.006
Composition ratio	9381	1743	1694	1707	2053	2184			
Hospital-acquired BSI	6901 (73.6%)	1279 (73.4%)	1202 (71.0%)	1228 (71.9%)	1528 (74.4%)	1664 (76.2%)	2.357	0.018*	0.031
Community-acquired BSI	2480 (26.4%)	464 (26.6%)	492 (29.0%)	479 (28.1%)	525 (25.6%)	520 (23.8%)	−2.357	0.018*	−0.031

*Z* value and *P* value were obtained by R Programming Language Cochran–Armitage trend test (CATT package)

*BSI*, bloodstream infection, *R*, Pearson correlation coefficient, ‰, episodes per 1000 adult-hospitalized patients

^*^Bold font means that the Cochran–Armitage trend test was statistically *P* < 0.05 and the dynamic time trend was significant

### Species distribution and dynamic trends

**Fig. 2:**
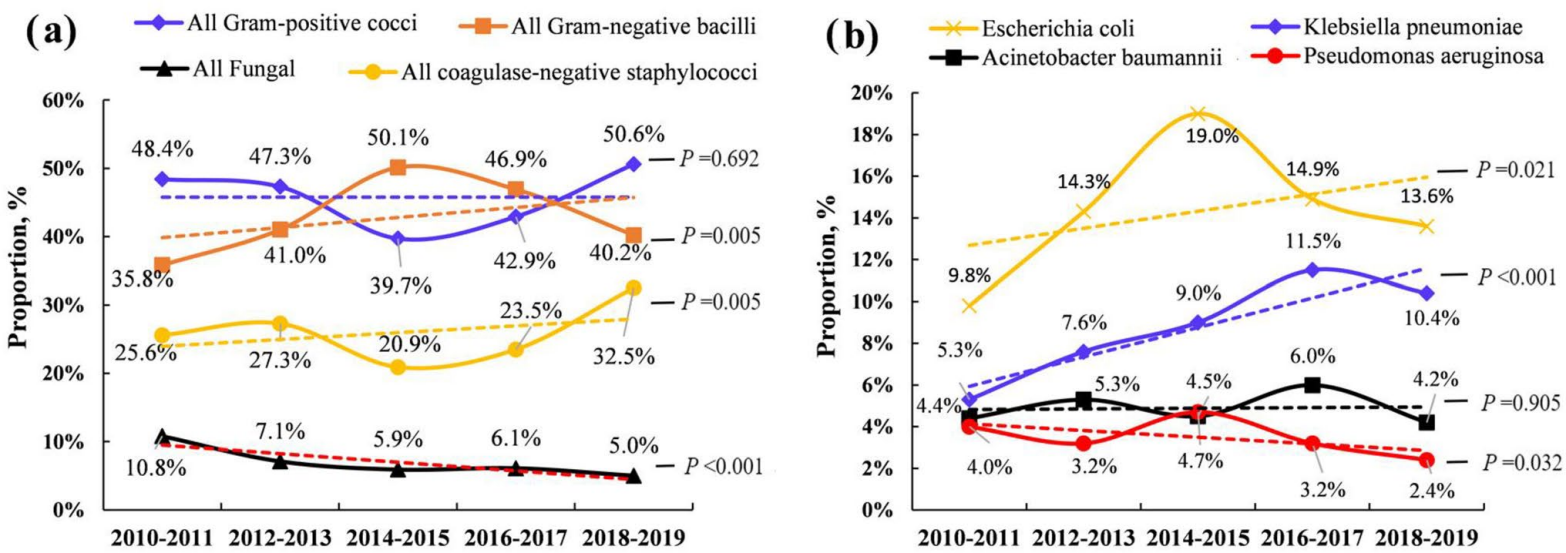
The species distribution in the 9381 episodes of bloodstream infection (BSI) and their dynamic time trends. Among the 9381 episodes of BSI, from 2010 to 2019, the direction of the dynamic trends of the proportions of Gram-negative bacilli (GNB), Coagulase-negative *staphylococcus* (CoNS), *Escherichia coli* and *Klebsiella pneumoniae* were increase, while both the proportions of *Pseudomonas aeruginosa* and all Fungal had decreased. In addition, both the proportions of all Gram-positive cocci (GPC) and *Acinetobacter baumannii* had not changed significantly

The species distribution and dynamic trends are presented in Table 2 and Fig. 2. Among the 9381 episodes of BSI, 93.1% (8737/9381) were bacteremia and 6.9% (644/9381) were fungemia. As shown in Fig. 2a, the proportion of fungemia decreased significantly from 10.8 to 5.0% for all species over the 10 years of the study (time trend *P* < 0.001, *R* = − 0.070). All species in fungemia decreased, regardless of whether they were Candida (6.1%, 573/9381) or non-Candida (0.8%, 71/9381). Both time trends were statistically significantly (*P* < 0.001).

**Table 2:** The species distribution, antimicrobial resistance and their dynamic trends in BSI

	Total (9381, 100%)	2010–2011 (n = 1743)	2012–2013 (n = 1694)	2014–2015 (n = 1707)	2016–2017 (*n* = 2053)	2018–2019 (*n* = 2184)	*Z* value	*P* value	*R*
Bacteremia	8737 (93.1%)	1555 (89.2%)	1574(92.9%)	1606 (94.1%)	1927 (93.9%)	2075 (95.0%)	5.325	< 0.001*	0.070
All Gram-positive cocci	4310 (45.9%)	843 (48.4%)	802(47.3%)	678 (39.7%)	881 (42.9%)	1106 (50.6%)	0.397	0.692	0.009
* Enterococcus faecium*	524 (5.6%)	66 (3.8%)	97 (5.7%)	109 (6.4%)	113 (5.5%)	139 (6.4%)	2.291	0.022*	0.030
* Staphylococcus aureus*	333 (3.5%)	58 (3.3%)	63 (3.7%)	65 (3.8%)	79 (3.8%)	68 (3.1%)	−0.247	0.805	−0.003
* Enterococcus faecalis*	176 (1.9%)	25 (1.4%)	28 (1.7%)	40 (2.3%)	48 (2.3%)	35 (1.6%)	0.699	0.484	0.009
All CoNS	2459 (26.2%)	447 (25.6%)	463(27.3%)	357 (20.9%)	483 (23.5%)	709 (32.5%)	2.823	0.005*	0.037
* Staphylococcus hominis*	821 (8.8%)	37 (2.1%)	122 (7.2%)	155 (9.1%)	192 (9.4%)	315 (14.4%)	10.394	< 0.001*	0.136
* Staphylococcus epidermidis*	678 (7.2%)	40 (2.3%)	73 (4.3%)	123 (7.2%)	184 (9.0%)	258 (11.8%)	10.026	< 0.001*	0.131
* Staphylococcus haemolyticus*	204 (2.2%)	18 (1.0%)	31 (1.8%)	41 (2.4%)	47 (2.3%)	67 (3.1%)	3.387	< 0.001*	0.044
* Staphylococcus capitis*	131 (1.4%)	2 (0.1%)	20 (1.2%)	21 (1.2%)	37 (1.8%)	51 (2.3%)	4.725	< 0.001*	0.062
All Gram-negative bacilli	4015 (42.8%)	624 (35.8%)	694(41.0%)	856 (50.1%)	963 (46.9%)	878 (40.2%)	2.821	0.005*	0.037
* Escherichia coli*	1339 (14.3%)	170 (9.8%)	242(14.3%)	324 (19.0%)	305 (14.9%)	298 (13.6%)	2.302	0.021*	0.030
* Klebsiella pneumoniae*	839 (8.9%)	93 (5.3%)	129 (7.6%)	153 (9.0%)	236 (11.5%)	228 (10.4%)	5.292	< 0.001*	0.069
*Acinetobacter baumannii*	457 (4.9%)	76 (4.4%)	89 (5.3%)	77 (4.5%)	124 (6.0%)	91 (4.2%)	0.120	0.905	0.002
*Pseudomonas aeruginosa*	323 (3.4%)	70 (4.0%)	55 (3.2%)	81 (4.7%)	65 (3.2%)	52 (2.4%)	−2.140	0.032*	−0.028
Fungemia	644 (6.9%)	188 (10.8%)	120 (7.1%)	101 (5.9%)	126 (6.1%)	109 (5.0%)	−5.325	< 0.001*	−0.070
Candida	573 (6.1%)	146 (8.4%)	107 (6.3%)	95 (5.6%)	119 (5.8%)	106 (4.9%)	−3.409	< 0.001*	−0.045
Non-Candida	71 (0.8%)	42 (2.4%)	13 (0.8%)	6 (0.4%)	7 (0.3%)	3 (0.1%)	−6.118	< 0.001*	−0.080
Antimicrobial resistance of Bacterial^#^	6224	816	941	1298	1470	1699			
Multidrug resistance	4257 (68.4%)	432 (52.9%)	472(50.2%)	1085 (83.6%)	1106 (75.2%)	1162 (68.4%)	7.841	< 0.001*	0.133
Sensitive	1967 (31.6%)	384 (47.1%)	469(49.8%)	213 (16.4%)	364 (24.8%)	537 (31.6%)	−7.841	< 0.001*	−0.133

Multidrug resistance (MDR) was defined as acquired non-susceptibility to at least one agent in three or more antimicrobial categories

*Z* value and *P* value were obtained by R Programming Language Cochran–Armitage trend test (CATT package)

*BSI*. bloodstream infection; *CoNS*, coagulase-negative staphylococci; *R*, Pearson correlation coefficient

^*^Bold font means that the Cochran–Armitage trend test was statistically *P* < 0.05 and the dynamic time trend was significant

^**#**^Since the antimicrobial resistance test results of 2513 (28.8%, 2513/8737) bacteria were lacked in the database, only 6224 bacteria were described in the table

Among the species in the BSI, 45.9% (4310/9381) were Gram-positive cocci (GPC), and 42.8% (4015/9381) were Gram-negative bacilli (GNB). Coagulase-negative staphylococcus (CoNS) accounted for the majority of GPC (26.2%, 2459/9381), and the four most common species were *Staphylococcus hominis* (8.8%), *Staphylococcus epidermidis* (7.2%), *Enterococcus faecium* (5.6%) and *Staphylococcus aureus* (3.5%). The four most common species of GNB were *Escherichia coli* (14.3%), *Klebsiella pneumoniae* (8.9%), *Acinetobacter baumannii* (4.9%) and *Pseudomonas aeruginosa* (3.4%).

From 2010 to 2019, the composition ratios of these species in BSI exhibited some dynamic changes over time. As shown in Table 2 and Fig. 2, the proportion of GNB increased from 35.8 to 40.2% (time trend *P* = 0.005). Although GPC exhibited no significant change (time trend *P* = 0.692), as the most common species group in GPC, the proportion of CoNS had increased from 25.6 to 32.5% (time trend *P* = 0.005, *R* = 0.037). The proportions of *Escherichia coli* (9.8–13.6%) and *Klebsiella pneumoniae* (5.3–10.4%) in the GNB group increased significantly over the 10 years of the study. However, the proportion of *Pseudomonas aeruginosa* decreased significantly from 4.0 to 2.4% (time trend *P* = 0.032, *R* = − 0.028). Some species did not significantly change, such as *Staphylococcus aureus* (3.3–3.1%, time trend *P* = 0.805) and *Acinetobacter baumannii* (4.4–4.2%, time trend *P* = 0.905), which are two important pathogenic bacteria in the clinic.

### Antimicrobial susceptibility of bacteria and dynamic trends

**Fig. 3:**
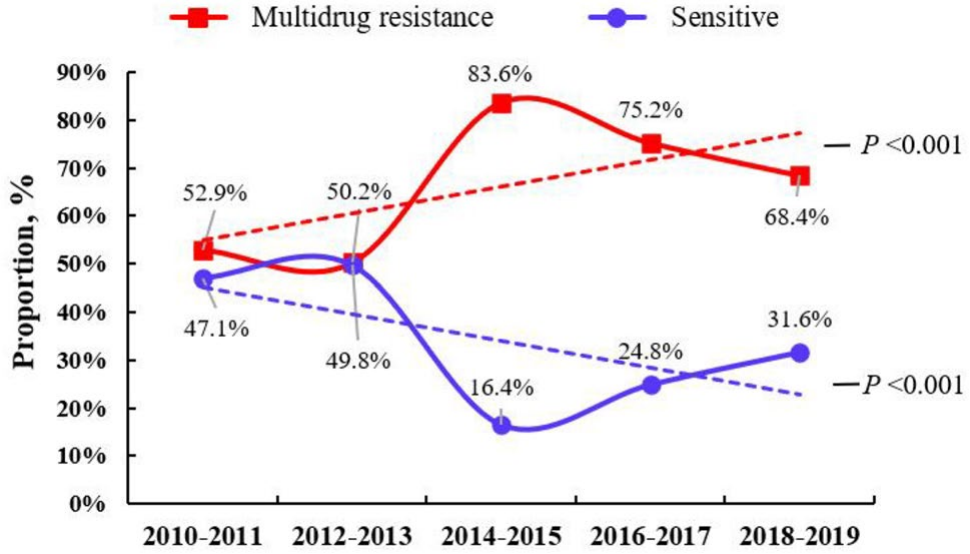
The multidrug resistance of bacteria in the 6224 episodes of bacteremia and their dynamic trends, and the results showed that the proportion of multidrug-resistant bacteria gradually significantly increased from 52.9 to 68.4% (time trend *P* < 0.001)

Although the RT-NISS system screened the antimicrobial susceptibility of 8737 species in bacteremia, only 6224 species (71.2%, 6224/8737) had complete data related to antimicrobial susceptibility to evaluate multidrug resistance, and 2513 species (28.8%, 2513/8737) lacked all or part of the data related to antimicrobial susceptibility. As shown in Table 2, 68.4% (4257/6224) of the 6224 species were resistant to at least three types of antibiotics and were considered multidrug resistant and 31.6% (1967/6224) were still sensitive to common antibiotics. However, as showed in Table 2 and Fig. 3, the proportion of drug-sensitive bacteria gradually decreased from 47.1 to 31.6% from 2010 to 2019, and the proportion of multidrug-resistant bacteria gradually increased from 52.9 to 68.4%. Both of these time trends were statistically significantly (time trend *P* < 0.001).

## Discussion

Bloodstream infection (BSI) is characterized by high mortality and multidrug resistance worldwide [15, 16]. The dynamic changes in species distribution and antimicrobial susceptibility are important clinical evidence for early empirical antimicrobial therapy of BSI, and these factors were the emphasis of the present study.

With the assistance of the highly sensitive and specific prospective real-time nosocomial infection surveillance system (RT-NISS) in our hospital, we identified 9381 episodes of BSI out of 1,437,927 adult-hospitalized patients over 10 years in CPLAGH, and we found that the total number of adult-hospitalized patients over two consecutive years increased from 2010–2011 to 2018–2019 (211,546 to 359,547 patients every 2 years) and that the corresponding number of the episodes of BSI increased from 1743 to 2184 episodes every 2 years. However, it was encouraging that the corresponding average proportion of BSI decreased significantly from 8.24 to 6.07 episodes per 1000 adult-hospitalized patients. The proportion was less than that in a Swedish county, which was from 9.45 to 15.46 per 1000 hospitalized patients from 2000 to 2013 [14]. This result might be due to the increased awareness of nosocomial infections and national action on infection control in China. The policies of Chinese government were implemented to promote the rational use of antibiotics from 2011 to 2014 in all Chinese hospitals [17]. In our hospital, the monthly utilization rate of antibiotics, the rate of antibiotic prophylaxis for type 1 incision surgery dropped from 63.7 to 41.8% and 91.9–49.3%, respectively. This decrease might be similar to that in the United States, in which the central line-associated BSI decreased by 46% between 2008 and 2013 as a result of national medical control [18].

However, we also found that the composition ratio of hospital-acquired BSI increased significantly annually (from 73.4 to 76.2%). This result was consistent with the increase in various hospital-acquired infections in recent years [19, 20]. In addition, the risk factors may be related to the risk factors for BSI and (or) hospital-acquired infections reported in many previous studies, such as ICU admission, the older age of hospitalized patients (aging population), prolonged hospital stay, leukocytopenia, acute myeloid leukemia and (or) increased use of invasive procedures including central venous catheters (CVC) [21–23]. However, more details of the risk factors require further statistical analysis of the decrease in hospital-acquired BSI in our hospital.

We found that the majority species in the 9381 episodes of BSI were bacteremia (93.1%) and that the average composition ratio over the two consecutive years gradually increased from 89.2% (2010–2011) to 95.0% (2018–2019) (time trend *P* < 0.001). Conversely, fungemia accounted for the minority of BSI (6.9%), and the average proportion decreased significantly from 10.8 to 5.0%. This result may be partially related to the increasing proportion of multidrug-resistant bacteria. Our data showed that the proportion of multidrug resistance of 6,224 bacteria increased significantly from 52.9 to 68.4% during the 10-year study period (time trend *P* < 0.001). The more multidrug-resistant bacteria increase the difficulty of antimicrobial therapy and prolong the antimicrobial therapy time period. However, the more multidrug-resistant bacteria do indeed seem to be related to the rise in bacteremia cases. We consider that among all types of nosocomial infections, such as lung infections and urinary tract infections, with the increase in multidrug-resistant bacteria and the prolonged the antimicrobial therapy time period may prompt and increase the chance that various kinds of pathogenic bacteria enter the blood. In this way, the multidrug-resistant bacteria might promote the rise in bacteremia cases. The increase of multidrug-resistant bacteria deserves our more attention, because multidrug-resistant bacteremia was a risk factor for mortality, just as reported by Ju MH et al. [24].

Although the composition ratio of bacteremia in the 9381 BSI increased over the 10-year study period, it did not mean that the composition ratio of all the species in bacteremia did not increase. Our data revealed that Gram-positive cocci (45.9%) and Gram-negative bacilli (42.8%) had a similar prevalence, and this result was different from the species distribution reported by the China Antimicrobial Surveillance Network (CHINET) in 2018 for all bacterial infections, including BSI and other bacterial infections (2018 CHINET report) [25]. The results of the CHINET report suggested that more Gram-negative bacilli (70%) were isolated than Gram-positive cocci (30%) and that the composition did not change obviously from 2005 to 2017 in China.

Notably, although the composition ratios of the Gram-positive cocci and Gram-negative bacilli were different with the 2018 CHINET report, the most common species of bacteria were consistent with the 2018 CHINET report. The top four most common Gram-negative species were *Escherichia coli* (14.3%), *Klebsiella pneumoniae* (8.9%), *Acinetobacter baumannii* (4.9%) and *Pseudomonas aeruginosa* (3.4%), which are identical to the CHINET report. Coincidentally, our data also revealed that the proportions of *Escherichia coli* (9.8–13.6%, time trend *P* = 0.021) and *Klebsiella pneumoniae* (5.3–10.4%, time trend *P* < 0.001) increased significantly, while the proportions of *Acinetobacter baumannii* (4.4–4.2%, time trend *P* = 0.905) and *Pseudomonas aeruginosa* (4.1–2.4%, time trend *P* = 0.032) both had decreased, similar to the CHINET report. In addition, because the composition ratio of Gram-negative bacteria (42.8%) in our study was lower than that in the CHINET report (70%) and fungi were not included in the CHINET report, the composition ratios of various species of Gram-negative bacteria were also different.

Among Gram-positive cocci, the most common species were coagulase-negative staphylococci (26.2%), and their proportion increased (25.6–32.5%, time trend *P* = 0.005) over the 10-year study period. *Staphylococcus aureus* accounted for only 3.5% in our study, but were the most common Gram-positive cocci in the CHINET report (9.0%). However, our result is not in conflict with the CHINET report because the CHINET report included various infections, not only BSI. Conversely, CoNS accounted for the minority of bacteria in the CHINET report (4.4%) because CoNS only originated from BSI. Therefore, the proportion of CoNS might be much higher only among BSI in CHINET report, and it was up to 26.2% in the present study.

In conclusion, we performed statistical analysis of the proportion, species distribution, and drug resistance of BSI as well as their dynamic changes over the past 10 years in one of the largest hospitals in China. We found that the proportion of BSI decreased dynamically over time and that the species distribution in BSI changed. The proportion of bacteria and multidrug resistance increased, and some species, such as *Klebsiella pneumoniae*, were obviously increased in bloodstream infection. Many of the increasing data presented above exhibited significant time trends and deserve clinical attention regarding infection control.

## Limitations

This study also had several limitations. First, the proportion of BSI might be underestimated in this study. A few pathogens, such as mycoplasma and chlamydia, are difficult to detect in conventional blood cultures, and some BSI were missed in false-negative blood cultures. In addition, some BSI were mistakenly excluded by the RT-NISS system used in our study because of the lack of typical clinical symptoms related to BSI (such as fever and increased inflammation makers). However, missing data might have had little effect on the results of this study, since the RT-NISS system had been used to screen BSI in a previous study and showed high sensitivity and specificity [11]. Second, the CSLS standards and the detection technology for blood cultures had been continuously updated and improved each year, which increased the positive detection rate of blood cultures and might have increased the proportion of BSI in recent years. Thus, this improvement in technology might have led to bias in the statistics of the dynamic time trend of the proportion over the 10 years. However, our data showed that the dynamic time trend of the proportion of BSI in adult-hospitalized patients decreased significantly. Therefore, the results of the statistical analysis of the time trend of the proportion could not be affected in this study. Last, the proportion and the species distribution of BSI were changing up and down in the past 10 years, since various related factors were also dynamic changing, such as the number, age, disease composition and severity of hospitalized patients. For this reason, all the *R* values in our study were relatively small and they were acceptable since the hospital had not undergone a particularly large adjustment. However, from an overall perspective, the proportion and species distribution of BSI were dynamically changing along certain trends. These trends deserved more attention from clinicians and researchers.

## Supplementary Information

Below is the link to the electronic supplementary material.

Supplemental Material.R: R Programming Language Cochran-Armitage trend test package (CATT) (r 2 kb)

## Data Availability

To avoid violating the privacy rules of the medical database in our hospital, all data involved in this study were not publicly available, but all data are available from the corresponding author for any reader if necessary.

## Abbreviations

BSI: Bloodstream infection
CHINET: China Antimicrobial Surveillance Network
HR: Hazard ratio
OR: Odds ratio
CPLAGH: Chinese People’s Liberation Army General Hospital
RT-NISS: Real-time nosocomial infection surveillance system
CA-BSI: Community-acquired BS
HA-BSI: Hospital-acquired BSI
CLSI: Clinical and Laboratory Standards Institute
GPC: Gram-positive cocci
GNB: Gram-negative bacilli
CoNS: Coagulase-negative staphylococcus
CVC: Central venous catheters
